# Comparison of perceptions of obesity among adults with central obesity with and without additional cardiometabolic risk factors and among those who were formally obese, 3 years after screening for central obesity

**DOI:** 10.1186/s12889-015-2544-1

**Published:** 2015-12-07

**Authors:** Corine den Engelsen, Rimke C. Vos, Mieke Rijken, Guy E. H. M. Rutten

**Affiliations:** Julius Center for Health Sciences and Primary Care, University Medical Center Utrecht, Huispostnr. STR.6.131, P.O. Box 85500, 3508 Utrecht, GA The Netherlands; NIVEL, Netherlands Institute for Health Services Research, Utrecht, The Netherlands

**Keywords:** Illness perceptions, Obesity, Prevention, Health behaviour, Cardiometabolic risk

## Abstract

**Background:**

Perceptions of illness are important determinants of health behaviour. A better understanding of perceptions of obesity might allow more effective interventions that challenge these perceptions through lifestyle modification programs. Although several studies have evaluated causal attributions with regard to obesity, other domains of illness perception, such as the perceived consequences of obesity and perceived controllability, have not yet been studied. The aim of the current study was to explore perceptions regarding causes, consequences, control, concerns and time course of obesity of centrally obese adults, with and without an elevated cardiometabolic risk and with or without weight loss, 3 years after screening for metabolic syndrome, and to compare these perceptions.

**Methods:**

Three groups were selected from a longitudinal study dependent on the baseline and 3-year follow-up profiles: individuals with central obesity and metabolic syndrome at both time points (‘persistent cardiometabolic-risk group’, *n* = 80), those with central obesity but without metabolic syndrome on either occasion (‘persistent obese group’, *n* = 63), and formerly obese individuals (‘improved cardiometabolic-risk group’, *n* = 49). Perceptions of obesity were assessed using an adapted version of the Brief Illness Perception Questionnaire (BIPQ, range 0–10). Chi-square and Kruskal-Wallis tests were performed to compare the ‘persistent cardiometabolic risk’ group with the other two groups with regard to patient characteristics and BIPQ scores.

**Results:**

Both males and females who improved their cardiometabolic risk perceived their obesity as shorter (median (IQR): 3.0 (4.0) vs. 6.0 (3.0), *p* < 0.001) and experienced greater personal control over their weight (7.0 (3.0) vs. 5.0 (3.0), *p* = 0.002) compared to those who did not improve. Females who improved their cardiometabolic risk experienced fewer identity and illness concerns, this was not found for males. Other scores did not differ between groups.

**Conclusion:**

Obese adults with an improved cardiometabolic risk profile felt greater personal control and considered their obesity to be of shorter duration. Persistence of central obesity with additional cardiometabolic risk factors had a larger impact on female than male participants with respect to identity and illness concerns. Whether discussing ‘personal control’ is a favourable element in lifestyle intervention should now be assessed in the setting of a controlled trial.

## Background

The prevalence of overweight and obesity is increasing. In 2009/2010, more than half of all Dutch adults aged 30–70 had a body mass index (BMI) ≥25.0 kg/m^2^ and 13 % of men and 14 % of women were obese (BMI ≥30.0 kg/m^2^) [1]. Corresponding numbers in other European Union countries and the United States are even higher [2, 3]. Due to the increasing prevalence of overweight and obesity, the number of people with hypertension, dyslipidaemia and an impaired glucose level is also rising. This will lead to a further increase in cardiovascular disease and type 2 diabetes.

Lifestyle modifications leading to modest weight loss can prevent cardiovascular disease (mainly due to the positive effects of weight loss on blood pressure and lipid profile) and diabetes [4–7]. However, changing health-related behaviour remains a challenge as the effectiveness of interventions in routine clinical care, outside a strictly controlled trial setting, has been disappointing or cannot be maintained in the long term [8–12]. This may be partly due to a sedentary lifestyle and an overabundance of high calorie foods, but may also relate to an individual’s perception of overweight and obesity. The Health Belief Model postulates that people need to experience a certain health threat before changing their behaviour [13]. Thus, people with overweight or obesity need to view their condition as serious and they should be aware of the associated health risks. In addition, overweight and obese people must feel in control in order to manage their weight, and they must believe that a specific behaviour will lead to a certain health outcome and that they will be able to adopt and maintain the desired behaviour – the so-called self-efficacy belief [14]. Self-efficacy beliefs significantly relate to a persons perceptions of control over their illness [15].

These various perceptions are combined in the Common Sense Model [16]. According to this model, people make sense of a health threat by developing their own cognitive and emotional perceptions or representations of that threat. These will determine how they cope with their health condition.

A number of studies have evaluated causal attributions with regard to obesity [17–20]. But other domains of illness perceptions, such as the perceived consequences of overweight and obesity and their perceived controllability, have not yet been studied. Likewise, it is not clear if persons with central obesity with and without an elevated cardiometabolic risk differ in their perceptions. It is possible that individuals with central obesity and elevated cardiometabolic risk factors attribute more symptoms to their overweight or obese condition, perceive more negative consequences and express more concern than persons with central obesity alone. Furthermore, those who suffered from obesity in the past but succeeded in losing weight might experience more personal control compared with people who failed to lose weight. Understanding these issues might allow more effective interventions to be developed that challenge these perceptions through lifestyle modification programs. Therefore, the aim of the current study was to explore the perceptions of individuals with screening-detected central obesity, with or without additional cardiometabolic risk factors. More specifically, we compared the perceptions of those who remained stable with those who improved their cardiometabolic risk profile during the follow-up period.

## Methods

### Study design and participants

Participants were selected from a large study that was designed to evaluate screening and follow-up in metabolic syndrome (MetS). The study included 11,862 individuals aged 20–70 years who were not previously diagnosed with diabetes, hypertension, dyslipidaemia or cardiovascular disease [21–24]. Screening took place between September 2006 and May 2007. The first stage was self-measurement of waist circumference with a tape measure mailed to the patient’s home. The results were sent to the research centre. A total of 1721 people with self-detected central obesity (waist circumference ≥88 cm in women and ≥102 cm in men) underwent further examinations at the research centre (waist circumference was verified by the research team; measured in duplicate, and the mean score of this was further used); 473 persons were diagnosed with MetS according to the National Cholesterol Education Program’s Adult Treatment Panel III (NCEP ATP III) [24, 25]. When cardiovascular risk factors were detected individuals were advised to contact their general practitioner (GP), advice that was followed by 72 % (increasing to 90 % after a reminder). No other intervention was performed [23]. All persons should have received usual care according to the guidelines ‘Cardiovascular risk management’ and ‘Type 2 Diabetes Mellitus’ of the Dutch College of General Practitioners [26, 27]. At follow-up, in November and December 2009, we compared perceptions with regard to obesity in three groups with central obesity at baseline (as determined by self-measured waist circumference). The first group had central obesity and MetS, both at baseline and at follow-up (the “persistent cardiometabolic risk group”). The second group was derived from a group of participants with central obesity at baseline and at follow-up but without MetS (the “persistent obesity group”). The third group consisted of people with central obesity and MetS at baseline, but no longer suffering from central obesity at follow-up (the “improved cardiometabolic risk group”). The study was approved by the Medical Ethical committee of the University Medical Center Utrecht.

### Measurements

Body weight, height, waist circumference and blood pressure were measured at the research centre, both at screening and follow-up. Venous blood samples were drawn after an overnight fast to determine fasting blood glucose, triglycerides and high-density lipoprotein (HDL) cholesterol. A detailed description of these measurements has been published previously [22, 24]. Data on prescribed cardiovascular medication at the time of follow-up were collected from the physician’s electronic medical records [23].

### Illness perceptions

At follow-up, participants completed an adapted version of the “Brief Illness Perception Questionnaire” (BIPQ [28]; Dutch version by Kaptein). The BIPQ includes eight items scored on an 11-point scale, ranging from 0 to 10 (Table 1). Five items assess cognitive illness representations: consequences, timeline, identity, personal control and treatment control. Two items assess emotional representations: concern and emotions. One item assesses coherence. A ninth open-ended response item assesses the patients’ causal representation by asking the patient to list in rank-order the three most important causal factors underlying their condition. The BIPQ has proven to be a reliable and valid measure of illness perceptions in a variety of patient populations [28]. In our adapted (not validated) version, we replaced the word ‘disease’ with the word ‘overweight’ in all items, similar to disease-specific versions of the more extended IPQ-R [http://www.uib.no/ipq].Furthermore, because obesity might be considered more a risk factor than a disease, we assumed that individuals might not fully understand the coherence item, and that the item on treatment control might not be considered applicable by all respondents. We therefore removed these two items. In place of these we added two items to gain more insight into outcome expectations, one of which referred to perceived control by physical activity and the second to perceived control by means of diet (Table 1). Since the original BIPQ items do not constitute one (or more) overarching scale(s), the scores of the six BIPQ items we included could still be compared with the scores from other patient populations.

**Table 1:** Adapted version of the Brief Illness Perception Questionnaire (BIPQ)

Question		Evaluated perception domain	Implication of a higher score
1	How much does your overweight affect your life?	Consequences: beliefs about the impact of the condition on physical, social and psychological well-being	Greater perceived influence of the condition upon life
2	How long do you think your overweight will continue?	Timeline: beliefs about the expected duration of the condition	A stronger belief in a chronic time course
3	How much control do you feel you have over your overweight?	Curability/controllability, personal control: beliefs about whether the condition can be cured or kept under control through self-management behaviour	Greater perceived personal control over the condition
4	How much do you think physical activity can help to improve your overweight?	Curability/controllability, behavioural control: beliefs about whether the condition can be cured or kept under control through physical activity	Greater perceived control over the condition by physical activity
5	How much do you experience symptoms from your overweight?	Identity: beliefs about the condition’s label and associated symptoms	The patient perceives more symptoms as a result of the condition
6	How concerned are you about your overweight?	Concern: evaluates to what extent concern is experienced	Greater feelings of concern about the condition
7	How much do you think a modification of your diet pattern can help improving your overweight?	Curability/controllability, behavioural control: beliefs about whether the condition can be ured or kept under control through diet	Greater perceived control over the condition by diet
8	How much does your overweight affect you emotionally? (e.g. does it make you angry, scared, upset or depressed?)	Emotional representations: beliefs about the affective responses associated with the illness	A stronger emotional response to the condition
9	Please list in rank-order the 3 most important factors that you believe caused your overweight.	Causal attribution: beliefs about factors or conditions to have caused the illness	

### Statistical analysis

To explore patient characteristics and illness perceptions of the three study groups, we computed descriptive statistics. Categorical variables are reported as numbers and percentages, continuous variables as means with standard deviations or median with inter quartile range (IQR) for non-normally distributed variables. Chi-square tests and the Kruskal-Wallis test were performed to compare the ‘persistent cardiometabolic risk’ group with the other two groups with regard to patient characteristics and BIPQ scores. A *p*-value < 0.05 was considered significant.

To identify potential confounders of the association between cardiometabolic risk level and obesity perceptions, we evaluated associations between socio-demographic characteristics and the BIPQ score for each domain. Socio-demographic characteristics that differed (*p* <0.05) between groups and that also showed a significant association with (at least one of) the perceptions of obesity, were considered as potential confounders. For gender, stratified analyses were performed.

Spearman correlation coefficients (r) were computed to assess associations between the various illness perception domains within each of the three groups.

The open answers to question nine (the factors that people believed to be important causes of their overweight) were classified into behavioural factors (diet and physical activity), biological factors (e.g. genetics, hormones), psychological factors (e.g. stress, depression) and other factors. Differences in frequency of each of these factors (lifestyle, biological, psychological or other) between the groups were tested using Chi-square tests.

Analyses were performed using the Statistical Package of Social Sciences (SPSS) version 20.0 (SPSS (IBM), Chicago, IL, USA).

## Results

A total of 406 of the 473 participants with screen-detected MetS were eligible for follow-up measurements (reasons for ineligibility were change of address or a critical illness) and were invited to participate, of whom 194 agreed. For the purpose of this study, we selected only those participants who continued to fulfil the central obesity and MetS criteria at follow-up (persistent cardiometabolic risk group; *n* = 80), and those who no longer had central obesity (improved cardiometabolic risk group; *n* = 49). In addition, 144 participants with central obesity but no other MetS components at baseline were invited for the follow-up examinations, of whom 88 agreed to participate. Of these, 63 had maintained their centrally obese phenotype (the persistent obesity group), of whom five (four females and one male) had developed MetS during follow-up, although none required antihypertensive, glucose lowering or lipid lowering medication (persistent obesity group) (Table 2). The ‘improved cardiometabolic risk’ group did not differ significantly from the ‘persistent cardiometabolic risk’ group with regards to age, gender, education level and ethnicity. Individuals in the latter group were significantly older and more likely to be male compared with the ‘persistent obesity’ group. Mean levels of BMI, blood pressure, triglycerides and fasting glucose were all significantly higher - or in case of HDL cholesterol, significantly lower - in the ‘persistent cardiometabolic risk’ group compared to the ‘persistent obesity’ group at the time of measurement of illness perceptions.

**Table 2:** Characteristics of 3 study groups

		Baseline			3 year follow-up		
		Persistent cardiometabolic risk group (*n* = 80)	Persistent obesity group (*n* = 63)	Improved cardiometabolic risk group (*n* = 49)	Persistent cardiometabolic risk group (*n* = 80)	Persistent obesity group (*n* = 63)	Improved cardiometabolic risk group (*n* = 49)
Gender (% male)		52.5	11.1^***^	65.3			
Ethnicity (% Western European)		95.0	98.4	100.0			
Higher education level (%)		28.7	27.0	30.6			
Age (years)		49.4 (9.6)	45.5 (9.2)	49.7 (10.9)	52.8 (10.0)	48.9 (9.2)^**^	53.2 (10.8)
BMI (kg/m^2^)		31.0 (3.6)	28.8 (3.3)	28.4 (2.1)	30.7 (3.7)	29.3 (3.4)^**^	25.9 (2.5)^***^
Waist circumference (cm)	Men	112.8 (10)	106.5 (4)	105.5 (5)	109.3 (12.2)	109.1 (5.3)	99.1 (3.8)^***^
	Women	101.8 (10)	95.3 (8)	92.0 (14)	98.9 (8.8)	95.2 (8.8)	82.0 (14.8)^***^
Blood pressure (mmHg)	Systolic	145.9 (17.3)	119.9 (5.9)	145.8 (13.5)	137.9 (14.8)	122.3 (8.4)^***^	133.6 (10.9)
	Diastolic	89.6 (7.6)	75.5 (4.7)	88.4 (7.6)	83.9 (7.2)	75.6 (5.5)^***^	80.9 (7.9)
Triglycerides (mmol/L)		1.8 (6)	0.9 (0)	1.9 (1)	2.1 (0.9)	1.2 (0.5)^***^	1.5 (1.0)^***^
HDL cholesterol (mmol/L)	Men	1.0 (0.4)	1.3 (0.4)	1.5 (0.7)	1.1 (0.4)	1.2 (0.4)	1.2 (0.5)
	Women	1.2 (0.3)	1.6 (0.5)	1.5 (0.6)	1.2 (0.2)	1.5 (0.5)^***^	1.4 (0.6)^**^
Fasting glucose (mmol/L)		5.2 (1.2)	4.8 (0.5)	4.9 (0.9)	5.5 (1.1)	5.0 (0.5)^***^	5.0 (0.8)^**^

Data are reported as mean (standard deviation) for age, BMI and blood pressure, as median (inter quartile range) for waist circumference, triglycerides, HDL cholesterol and fasting glucose or percentage

P-value for difference at 3 year follow-up between persistent cardiometabolic risk and persistent obesity groups: ^*^ *p* < 0.05; ^**^ *p* < 0.01; ^***^ *p* < 0.001

P-value for difference at 3 year follow-up between persistent and improved cardiometabolic risk groups: ^*^ *p* < 0.05; ^**^ *p* < 0.01; ^***^ *p* < 0.001

*BMI* body mass index, *HDL cholesterol* high-density lipoprotein cholesterol

### Difference in illness perceptions between groups

Compared to the ‘improved cardiometabolic risk’ group, both the ‘persistent cardiometabolic risk’ and ‘persistent obesity’ groups differed significantly with regard to ‘timeline’; the latter two groups considered their condition to have a more or less chronic time course (median (IQR): 3 (4) vs. 6 (3) and 7 (3), both <0.01), with mild to modest consequences (median (IQR): 3 (4.5) vs.4 (3) and 4 (4), *p* = 0.03). They experienced few symptoms due to their overweight (median (IQR): 1 (4) vs. 3 (4.75) and 2.5 (4.25), both *p* = 0.03). In general, individuals in the ‘persistent cardiometabolic risk’ and ‘persistent obesity’ group believed that physical activity and diet could have a positive effect on their body weight (open-ended question). Their score on perceived personal control, however, shows that they were not convinced that they could influence their condition themselves, indicating relatively low self-efficacy beliefs (median (IQR): 7 (3) vs. 5 (3) and 5 (2.25), both *p* < 0.01).

Stratification for gender did not influence these findings. However, gender did have an effect on identity perception and on illness concerns. In the ‘improved cardiometabolic risk’ group only females showed fewer identity and illness concerns than their counterparts in the ‘persistent cardiometabolic risk’ group (Table 3).

**Table 3:** Mean BIPQ scores in the 3 study groups stratified by gender

	Male			Female		
	Persistent cardiometabolic risk group (*n* = 80)	Persistent obesity group (*n* = 63)	Improved cardiometabolic risk group (*n* = 49)	Persistent cardiometabolic risk group (*n* = 80)	Persistent obesity group (*n* = 63)	Improved cardiometabolic risk group (*n* = 49)
Consequence	4.0 (3.0)	3.5 (3.5)	3.0 (5.0)	4.0 (4.25)	4.0 (4.0)	2.0 (4.5)
Timeline	6.0 (3.25)	6.5 (3.5)	4.0 (2.75)^***^	7.0 (3.5)	7.0 (2.75)	1.0 (2.5)^***^
Personal control	5.0 (3.0)	5.0 (0.75)	7.0 (3.75)^*^	5.0 (3.0)	5.0 (2.75)	8.0 (4.0)^*^
Behavioural control – physical activity	8.0 (4.0)	6.5 (5.5)	8.0 (3.0)	7.0 (2.0)	7.0 (3.0)	8.0 (3.0)
Identity	3.0 (4.0)	2.5 (3.25)	2.0 (4.0)	3.0 (5.0)	2.5 (5.0)	0.0 (1.5)^**^
Illness concern	4.0 (5.0)	3.5 (4.25)	5.0 (4.0)	4.0 (4.0)	4.0 (3.0)	2.0 (3.0)^***^
Behavioural control - diet	8.0 (3.0)	8.0 (2.75)	7.0 (3.0)	7.0 (3.5)	7.0 (4.0)	8.0 (4.0)
Emotional representation	2.0 (3.0)	2.5 (4.75)	2.0 (4.75)	3.0 (5.0)	3.5 (6.0)	2.0 (3.5)

Data are reported as median (inter quartile range), range 0–10

P-value for difference between persistent cardiometabolic risk and persistent obesity groups, unadjusted: ^*^ *p* < 0.05; ^**^ *p* < 0.01; ^***^ *p* < 0.001

P-value for difference between persistent and improved cardiometabolic risk groups, unadjusted ^*^ *p* < 0.05; ^**^ *p* < 0.01; ^***^ *p* < 0.001

### Correlations of illness perceptions

In all three groups (Table 4), high correlations (*r* ≥ 0.62, *p* < 0.001) were found for the consequences, identity and concern items. Scores on the two behavioural control items (diet and exercise) were also correlated. For the ‘persistent cardiometabolic risk’ and the ‘improved cardiometabolic risk’ groups, but not the ‘persistent obesity’ group, scores on the behavioural control items significantly correlated with the personal control score. For both the ‘persistent cardiometabolic risk’ and ‘persistent obesity’ groups, scores on the emotional representation item correlated highly with scores on the consequences, identity and concern items. For the ‘improved cardiometabolic risk’ group, these correlations were weaker (*r* ≤ 0.45) and not all were significant.

**Table 4:** BIPQ scale correlations (r) in the 3 study groups

	BIPQ scale	Timeline	Personal control	Behavioural control – physical activity	Identity	Illness concern	Behavioural control - diet	Emotional representation
Persistent cardiometabolic risk group (*n* = 80)	Consequence	0.29**	0.15	0.10	0.71**	0.68**	0.15	0.61**
	Timeline		0.05	0.08	0.36**	0.36**	0.10	0.21
	Personal control			0.23*	−0.01	0.07	0.23*	0.02
	Behavioural control – physical activity				−0.03	0.14	0.43**	−0.04
	Identity					0.67**	0.21	0.58**
	Illness concern						0.24*	0.51**
	Behavioural control - diet							0.11
Persistent obesity group (*n* = 63)	Consequence	−0.07	−0.20	0.29*	0.62**	0.66**	0.35**	0.60**
	Timeline		−0.20	−0.04	−0.02	−0.10	−0.08	−0.01
	Personal control			0.03	−0.19	−0.08	−0.01	−0.07
	Behavioural control – physical activity				0.32*	0.30*	0.55**	0.28*
	Identity					0.67**	0.20	0.56**
	Illness concern						0.34**	0.63**
	Behavioural control - diet							0.52**
Improved cardiometabolic risk group	Consequence	0.42**	0.33**	0.15	0.63**	0.75**	0.35*	0.36*
	Timeline		−0.18	−0.06	0.61**	0.61**	−0.09	0.18
	Personal control			0.50**	0.12	0.22*	0.32*	0.07
	Behavioural control – physical activity				0.07	0.32*	0.42**	0.09
	Identity					0.62**	0.15	0.30*
	Illness concern						0.20	0.45**
	Behavioural control - diet							0.32*

Spearman correlation coefficients

*Correlation is significant at the 0.05 level (2-tailed)

**Correlation is significant at the 0.01 level (2-tailed)

In both the ‘persistent cardiometabolic risk’ and ‘improved cardiometabolic risk’ groups, individuals who considered their condition to be more chronic had significantly higher scores on the consequences, identity and concern items; in the ‘persistent obesity’ group, timeline scores did not correlate with scores on any of the other items.

### Causal factors for obesity

The majority of the participants identified lifestyle factors as the most important causal factors for their (former) obesity, diet being more often mentioned than physical activity. Although this percentage was highest in the ‘improved cardiometabolic risk’ group, the difference compared to the other two groups was not significant. In the ‘persistent cardiometabolic risk’ and ‘persistent obesity’ groups, about one in five participants considered biological factors as contributors to their central obesity, while 7–10 % mentioned psychological factors (Table 5).

**Table 5:** Causes of obesity, as mentioned by participants

	Mentioned as reason (%)		
	Persistent cardiometabolic risk group *N* = 71	Persistent obesity group *N* = 59	Improved cardiometabolic risk group *N* = 42
Lifestyle	91.5	88.1	97.6
Diet	^a^77.5	^a^83.1	^a^85.7
Physical activity	^a^50.7	^a^44.1	^a^52.4
Lifestyle, not specified	^a^7.0	^a^1.7	^a^9.5
Biological	16.9	27.1	11.9
Genetics	^a^15.5	^a^15.3	^a^7.1
Other	^a^1.4	^a^11.9	^a^4.8
Psychological	7.0	10.2	7.1
Other	23.9	20.3	14.3

^a^Percentages of participants within the causal factor for obesity

## Discussion

The current study explored obesity-related perceptions amongst adults with central obesity with or without cardiometabolic risk factors, and amongst individuals with former central obesity. We expected that the persistency of central obesity, combined with the detection of additional cardiometabolic risk factors 3 years previously, would have had an impact on people’s perceptions of their obesity. However, we could not demonstrate that the presence of additional cardiometabolic risk factors goes together with the perception of obesity as a more serious condition. The relatively low scores on the consequences, identity and concern items indicate that neither group considered obesity to be very serious. While the idea that diet and physical activity can have a positive influence on weight was widely accepted by participants, the moderate score on perceived personal control suggests that obese individuals have little confidence in their own ability to control their body weight. However, both males and females with central obesity at baseline—the improved cardiometabolic risk group—scored higher on the personal control item than those in the ‘persistent cardiometabolic risk’ group, indicating that those individuals who succeed in losing weight experience more personal control than those who do not.

Persistence of central obesity with additional cardiometabolic risk factors had a greater impact on female than on male participants. Women who improved their cardiometabolic risk profile perceived their overweight as less identity restricting and less alarming than women without cardiometabolic improvements. These differences were not found for men.

Almost all participants acknowledged that lifestyle factors were important causes of their overweight or obesity. No correlation was found in the persistent central obesity group between behavioural control scores and personal control scores. This suggests that those who fail to lose body weight may believe that changing diet and physical activity patterns can help to reduce weight, but at the same time do not necessarily believe they can influence their own body weight. Those in the ‘persistent obesity’ group were unlikely to have received regular follow-up care by a practice nurse, dietician or GP because they did not show additional cardiometabolic risk factors. This might explain why participants in this group did not feel a high level of personal control over their condition. However, the group with persistent central obesity and additional risk factors also perceived low ‘personal control’, despite being advised to contact their GP. These findings suggest that persons with central obesity between the ages of 20 and 70 years, whether or not they have additional cardiometabolic risk factors or have consulted their GP in connection with this, would receive greater benefit from support that enhanced their self-efficacy beliefs than from more information about the role of a healthy diet and physical activity.

### Strengths and limitations

Because illness perceptions were determined only at follow-up, we could not assess causal relations between perceptions regarding obesity and past or present overweight/obesity. Perceptions might have been influenced by follow-up counselling in both the ‘persistent cardiometabolic risk’ and the ‘improved cardiometabolic risk’ groups. Likewise, the ‘persistent obesity’ group consisted predominantly of females and was 4 years younger on average than the other groups, which might have influenced results. This group may go on to develop features of MetS at a later stage, as indicated by mean waist circumference, systolic blood pressure, triglyceride and glucose levels, all of which increased after follow-up (in contrast to the other groups). During follow-up, five (four females and one male) of the 63 persons with persistent obesity did in fact develop MetS. In order to compensate for a possible gender difference, we analysed group illness perceptions separately for males and females. In addition, due to the relatively small group sizes, we may lack power to demonstrate clinically relevant differences in perceptions regarding obesity between the ‘persistent obesity’ and the ‘persistent cardiometabolic risk’ groups. Nevertheless, as an initial investigation, we believe that this study has produced interesting findings that deserve further examination in a larger study.

The final open answer BIPQ item assessed patients’ causal perceptions by asking them to list in rank-order the three most important causal factors for their excess body weight. Mentioning physical activity and diet as treatment options in previous questions might have biased the results in this section.

### Comparison with existing literature

We compared the perceptions of (former) centrally obese adults with the illness perceptions of adults with type 2 diabetes [28], and adults with hypertension [29]. Compared with a group of New Zealand type 2 diabetes patients (mean age 57.2 years, 52.9 % male) and a group of Portuguese hypertension patients (mean age 62 years, 41 % male), both our ‘persistent cardiometabolic risk’ and ‘persistent obesity’ groups had lower scores on the timeline, consequences, identity, and concern items; thus, they perceived their condition to be less threatening compared with the diabetes and hypertension patients. People with diabetes and hypertension, however, experienced more personal control over their condition. This may be due to the fact that both diabetes and hypertension can be controlled by medication.

Lifestyle factors were mentioned as important causal influences by approximately 90 % of the participants with persistent obesity both with and without additional cardiometabolic risk factors, and by 98 % of the former centrally obese adults, which is higher than any previously reported. Wang et al. studied a mixed sample of lean, overweight and obese people and asked them to what extent they believed either lifestyle factors or inheritance influenced obesity. Of the respondents, 72 % endorsed the belief that lifestyle behaviours have ‘a lot’ to do with causing obesity, whereas only 19 % indicated that inheritance has ‘a lot’ to do with causing obesity [20]. Hilbert et al. assessed causal attributions of obesity in obese people presenting for genetic testing and counselling. Sixty-one percent of the participating women and 73 % of the men endorsed behavioural attributions, whereas 86 % of women and 60 % of men endorsed genetic/biological attributions [18]. In both of these studies, however, answers regarding causal factors were structured, whereas we used an open-ended question. We suspect that our approach facilitates the expression of more spontaneous and deeply-based perceptions.

## Conclusion

Individuals with persistent central obesity, irrespective of additional cardiometabolic risk factors, seem to underestimate the seriousness of their condition and experience relatively low levels of personal control. Persistence of central obesity with additional cardiometabolic risk factors had a greater impact on female than on male participants. Women who improved their cardiometabolic risk profile perceived their overweight as less identity restricting and less alarming than women without cardiometabolic improvements. These differences were not found for men. Lifestyle interventions, especially for women with obesity - regardless of the presence of additional cardiometabolic risk factors - should therefore focus on increasing awareness of the serious implications of overweight or obesity, on boosting personal confidence in an ability to influence body weight and on enhancing a sense of personal control. Whether discussing ‘personal control’ is a favourable addition in a lifestyle intervention should now be assessed in the setting of a controlled trial.

## Abbreviations

BIPQ: Brief illness perception questionnaire
BMI: Body mass index
HDL: High-density lipoprotein
IQR: inter quartile range
MetS: Metabolic syndrome
NCEP ATP III: National Cholesterol Education Program’s Adult Treatment Panel III
